# Hydrocephalus after foramen magnum decompression for Chiari I malformation successfully treated with the aspiration of pseudomeningocele: a case report

**DOI:** 10.1186/s13256-019-2191-8

**Published:** 2019-08-06

**Authors:** Takashi Kobayashi, Naohisa Miyakoshi, Toshiki Abe, Kazuma Kikuchi, Eiji Abe, Yoichi Shimada

**Affiliations:** 1 Department of Orthopedic Surgery, Akita Kousei Medical Center, 1-1-1 Iijima, Nishifukuro, Akita, 011-0948 Japan; 2 Department of Orthopedic Surgery, Akita University Graduate School of Medicine, 1-1-1 Hondo, Akita, 010-8543 Japan

**Keywords:** Chiari I malformation, Foramen magnum decompression, Hydrocephalus, Pseudomeningocele, Case report

## Abstract

**Background:**

Pseudomeningocele or cerebrospinal fluid leakage is one of the most common complications of foramen magnum decompression with duraplasty for Chiari I malformation. Usually, cerebrospinal fluid leakage is treated with lumbar drainage and/or secondary suture. However, if hydrocephalus occurs, spinal drainage may cause brain herniation.

**Case presentation:**

A 54-year-old Japanese woman presented to our hospital with a 10-month history of bilateral finger extension weakness and clumsiness. Magnetic resonance imaging showed displacement of her cerebellar tonsils below the foramen magnum level, with syringomyelia presenting from the C4 to T8 level. Suboccipital craniectomy and C1 laminectomy with duraplasty were performed under general anesthesia. At 1 month after discharge, she again presented to our hospital due to severe headache and nausea. Magnetic resonance imaging of her cervical spine showed pseudomeningocele compressing her cerebellum and spinal cord. Magnetic resonance imaging of her brain also showed ventriculomegaly. Pseudomeningocele aspiration was performed, with 25 ml of fluid removed under X-ray control. Immediately after aspiration her headache and nausea decreased, and she reported improvement in her symptoms with increasing bilateral finger extension strength and decreasing bilateral upper extremity numbness at her 1-year follow-up.

**Conclusions:**

Although there is a considerable risk of meningitis with the aspiration procedure of pseudomeningocele, an aspiration procedure may be an easy and effective treatment option for postoperative hydrocephalus after suboccipital craniotomy with duraplasty in a patient treated for Chiari I malformation.

## Background

Pseudomeningocele or cerebrospinal fluid (CSF) leakage is one of the most common complications of foramen magnum decompression (FMD) with duraplasty for Chiari I malformation (CM-I) [1–4]. Usually, CSF leakage is treated with lumbar drainage and/or secondary suture [2]. However, if hydrocephalus occurs, spinal drainage may cause brain herniation [5].

## Case presentation

**Fig. 1:**
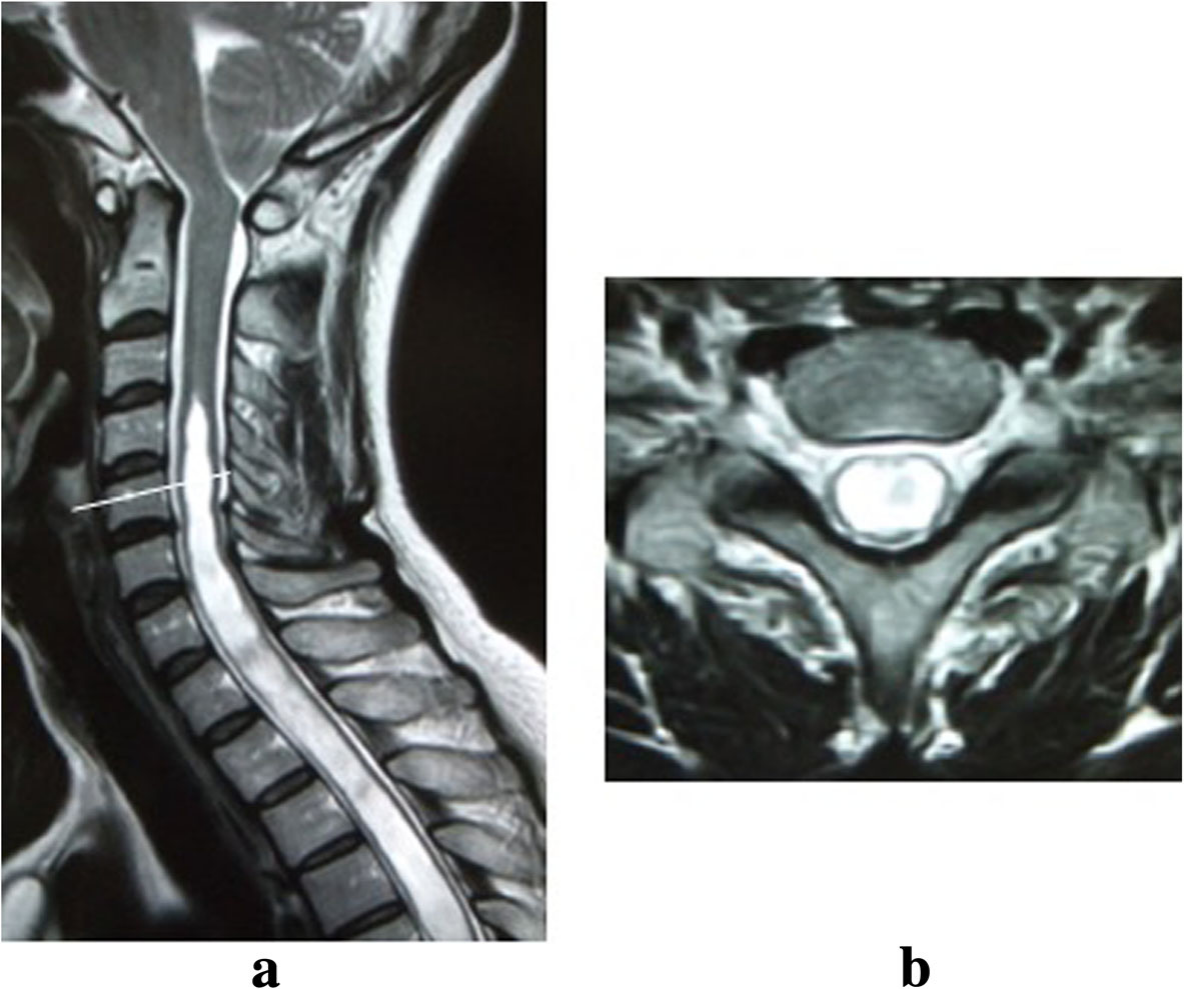
Preoperative cervical magnetic resonance imaging of a 54-year-old woman. **a** Sagittal image. The cerebellar tonsils are displaced below the level of the foramen magnum. There is a high T2-weighted signal observed in the cervical and thoracic cord. **b** Axial image at the C5 level. There is a high T2-weighted signal in the cervical cord

A 54-year-old Japanese woman presented to our hospital with a 10-month history of bilateral finger extension weakness and clumsiness. The chief complaint at her first visit was bilateral ulnar side numbness of her forearm and middle finger, ring finger, and little finger. There was atrophy of intrinsic muscles. She had no decline in her balance or ambulatory abilities. Magnetic resonance imaging (MRI) showed displacement of her cerebellar tonsils below the foramen magnum level, with syringomyelia presenting from the C4 to T8 level (Fig. 1). A surgical procedure was planned due to the progressiveness of her neurological conditions.

### Operation

Suboccipital craniectomy and C1 laminectomy with duraplasty were performed under general anesthesia. The craniotomy was 3 cm in size and rectangular. After opening the dura in a Y-shaped fashion, a pinhole unexpectedly opened in the arachnoid membrane. The dural graft used an absorbable artificial dural substitute (Seamdura, Gunze Limited Medical Division, Tokyo, Japan), which was carefully sewn into place using a running 5-0 nylon suture. After placement of an absorbable suture reinforcement felt sheet made of polyglycolic acid (Neoveil, Gunze Limited) for use as an absorbable artificial dural substitute interface, the surface was covered by fibrin glue. The total operative time was 3 hours 27 minutes, and there was little estimated blood loss.

### Postoperative course

**Fig. 2:**
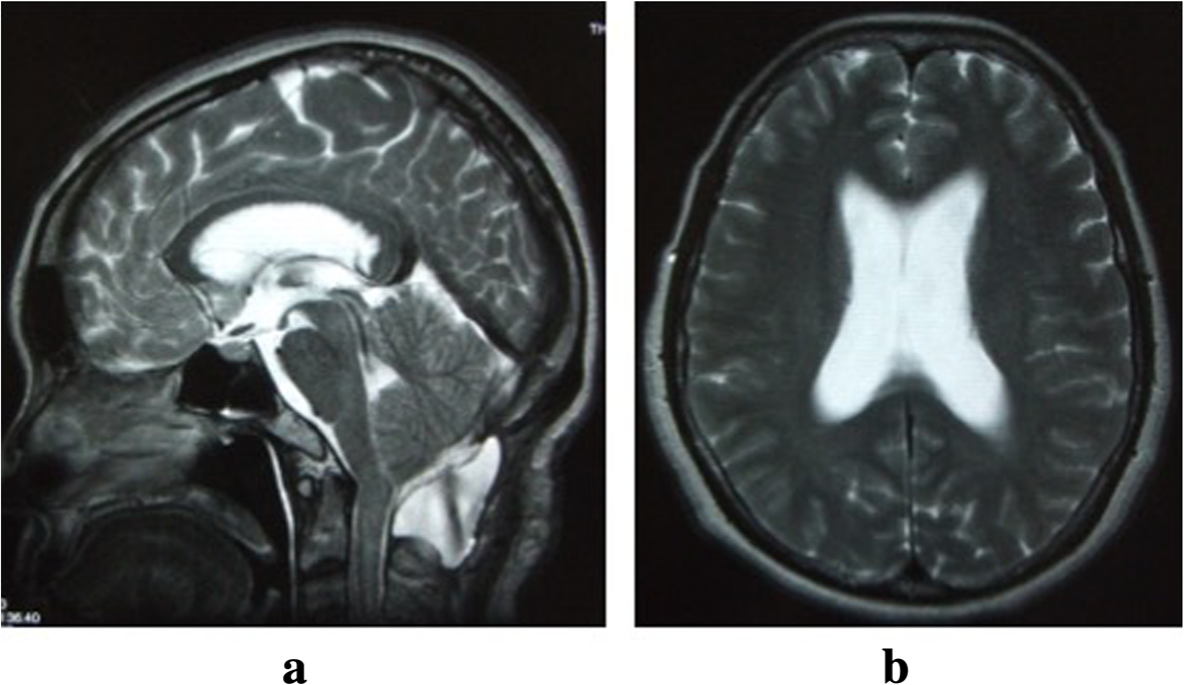
Magnetic resonance T2-weighted image of the brain. **a** Sagittal image at 2 months postoperative shows that the pseudomeningocele at the operation site is compressing the cerebellum and spinal cord. **b** Axial image at 2 months postoperative shows ventricle enlargement

**Fig. 3:**
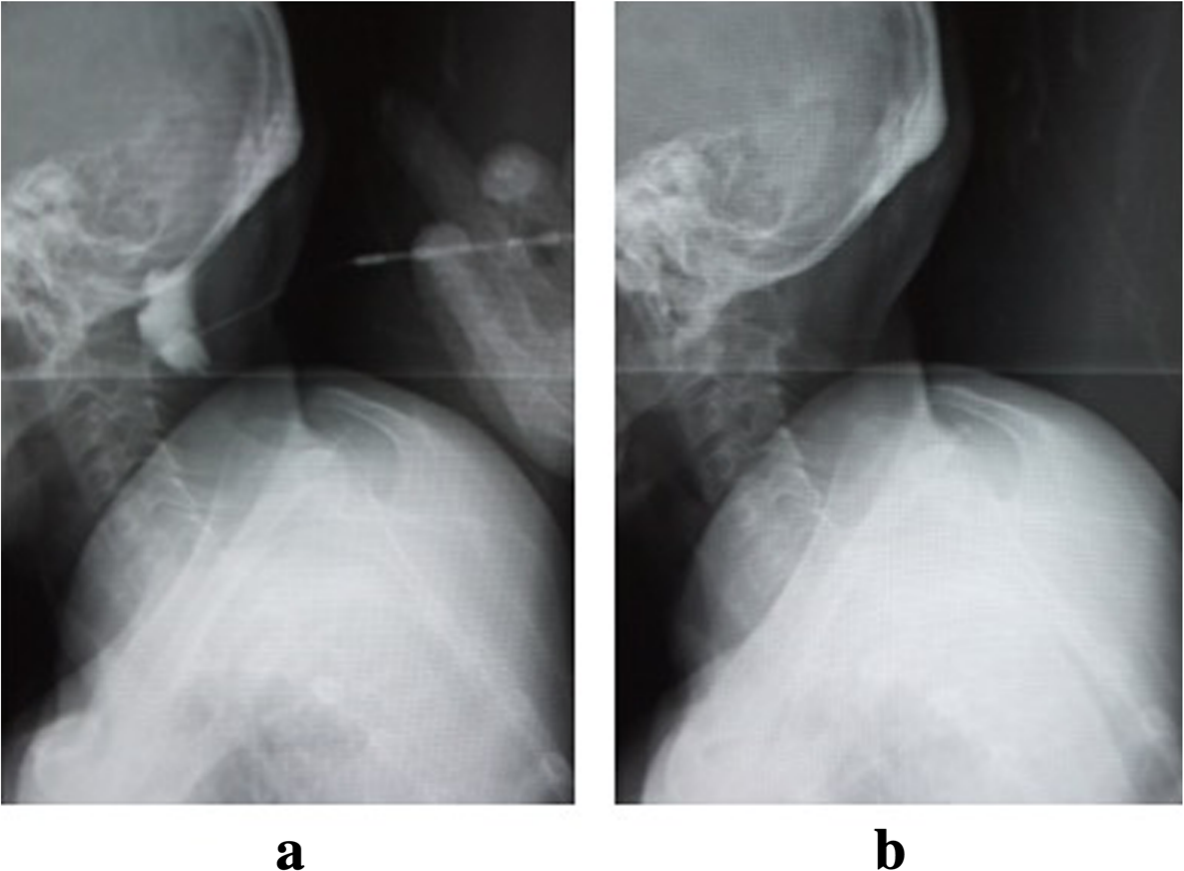
X-ray taken during the aspiration of the pseudomeningocele. **a** X-ray taken of the pseudomeningocele during the injection of contrast medium. **b** X-ray taken after aspiration of the contrast medium and spinal fluid

**Fig. 4:**
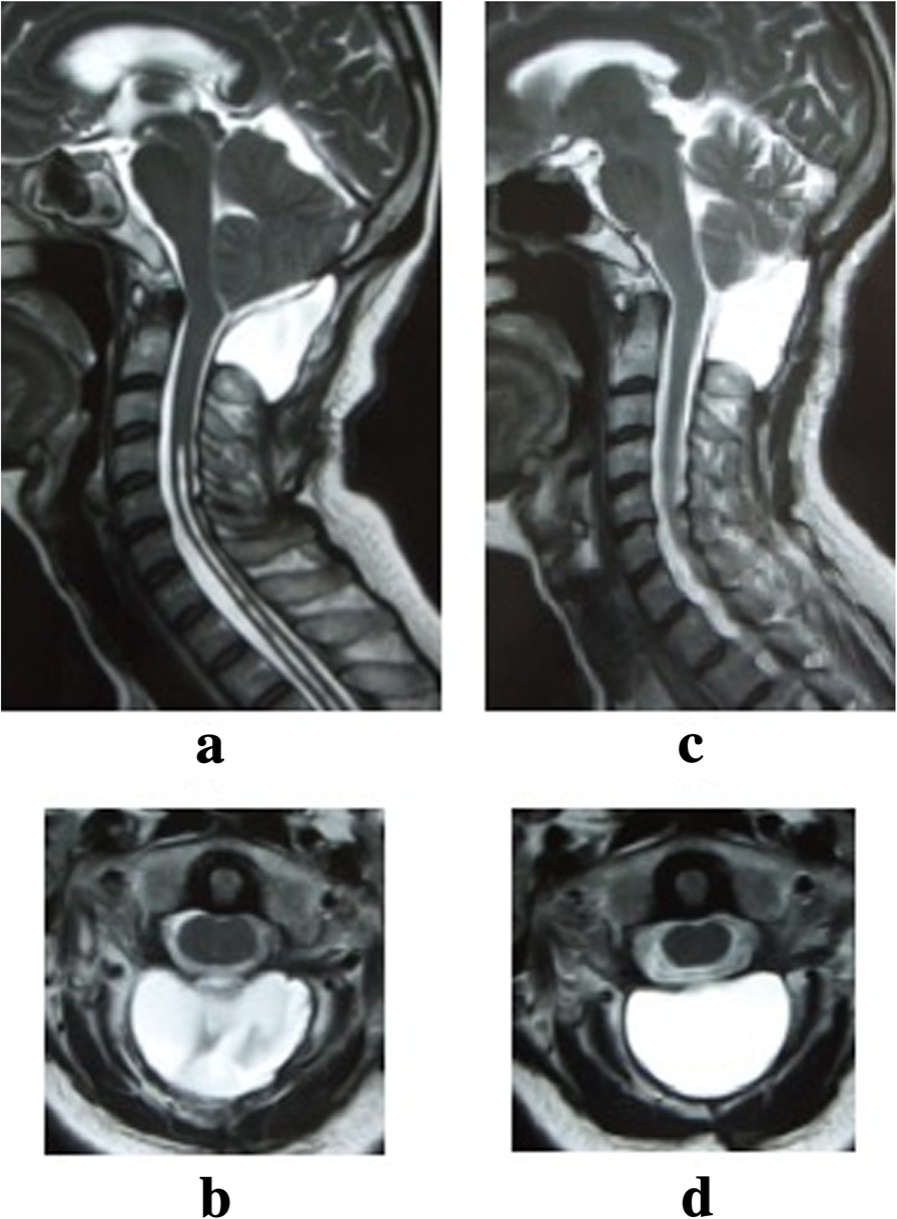
Magnetic resonance T2-weighted image of the cervical spine. **a** Sagittal image at 2 months postoperative. Although shrinkage of spinal syringomyelia is observed, the pseudomeningocele at the operation site is compressing the cerebellum and spinal cord. **b** Axial image at 2 months postoperative shows the pseudomeningocele at the surgical site. **c** Sagittal image at 4 months postoperative shows the size of the pseudomeningocele is getting smaller and the pseudomeningocele does not compress the cerebellum and spinal cord. **d** Axial image at 4 months postoperative shows there is a space around the spinal cord

Postoperative bleeding was 80 ml while under a zero vacuum pressure drain, which was removed at 2 days after surgery. Although our patient exhibited no new neurological deficits, she had a prolonged headache. At the time of her release at 1 month after the operation, she still had a slight headache. At 1 month after discharge, she again presented to our hospital due to severe headache and nausea. On examination, her wound was well healed and there was no evidence of CSF leakage or infection. There were also no cranial neurological symptoms observed. MRI of her cervical spine showed pseudomeningocele compressing her cerebellum and spinal cord. MRI of her brain also showed ventriculomegaly (Fig. 2). Pseudomeningocele aspiration with a 22-gauge hypodermic needle was performed, with 25 ml of fluid removed under X-ray control (Fig. 3). Immediately after aspiration, her headache and nausea decreased, and she was subsequently discharged at 3 weeks after aspiration. At 4 months after the operation, cervical spine MRI showed shrinkage of spinal syringomyelia and resolution of the stenosis at the level of the cerebellar tonsils (Fig. 4), although the pseudomeningocele remained. At her 1-year follow-up, she reported improvement in her symptoms with increasing bilateral finger extension strength and decreasing bilateral upper extremity numbness.

Institutional Review Board approval was obtained for this case report, and our patient gave written informed consent to publish this case and any accompanying images.

## Discussion and conclusion

Although pseudomeningocele aspiration should be restricted due to possible infection or its recurrence, we were able to successfully treat the myelomeningocele in this case using aspiration. The reason for our success may be because the arachnoid membrane defect was so small. Second, it also might be related to the fact that the symptomatic pseudomeningocele occurred at 2 months after surgery.

Hydrocephalus after FMD occurs in 2 to 8.7% of cases [4, 6, 7]. Although the definitive cause of hydrocephalus remains unknown, some reports have suggested it can be caused by infratentorial fluid collection [8, 9]. Treatment of symptomatic hydrocephalus normally involves drainage of CSF using a ventricular drain [8, 9]. However, since MRI showed the pseudomeningocele was compressing our patient’s cerebellum and spinal cord, we used aspiration of the pseudomeningocele to successfully treat our patient.

Although there is a considerable risk of meningitis with the aspiration procedure of pseudomeningocele, the aspiration procedure may be an easy and effective treatment option for postoperative hydrocephalus after suboccipital craniotomy with duraplasty in a patient treated for CM-I.

Although we could choose FMD without duraplasty to avoid pseudomeningocele, this procedure leads to an unreliable reduction in the symptoms compared with FMD with duraplasty [10]. When duraplasty is employed, no literature supports the superiority of either autologous or non-autologous grafts. However, autologous materials may be a good option because they are non-immunogenic, inexpensive, and capable of creating a watertight closure with the dura [11].

## Data Availability

Not applicable.

## Abbreviations

CM-I: Chiari I malformation
CSF: Cerebrospinal fluid
FMD: Foramen magnum decompression
MRI: Magnetic resonance imaging
